# Japanese living donor liver transplantation criteria for hepatocellular carcinoma: nationwide cohort study

**DOI:** 10.1093/bjsopen/zrae079

**Published:** 2024-08-02

**Authors:** Masahiro Ohira, Gaku Aoki, Yasushi Orihashi, Kenichi Yoshimura, Takeo Toshima, Etsuro Hatano, Susumu Eguchi, Taizo Hibi, Kiyoshi Hasegawa, Yuzo Umeda, Takuya Hashimoto, Yasushi Hasegawa, Shuji Nobori, Yasuhiro Ogura, Hiroyuki Nitta, Hiroto Egawa, Hidetoshi Eguchi, Yasutsugu Takada, Yoshihide Ueda, Mureo Kasahara, Shigeyuki Kawachi, Yuji Soejima, Katsutoshi Tokushige, Hiroaki Nagano, Hironori Haga, Takumi Fukumoto, Satoshi Mochida, Koji Umeshita, Hideki Ohdan, the Japanese Liver Transplantation Society, Shugo Mizuno, Satoshi Kuboki, Masayuki Otsuka, Takashi Kobayashi, Toshifumi Wakai, Teruhide Ishigame, Shigeru Marubashi, Masaaki Watanabe, Akinobu Taketomi, Shinichi Nakamuma, Shintaro Yagi, Taichi Wakiya, Keinosuke Ishido, Yu Sawada, Itaru Endo, Yuichi Goto, Toru Hisaka, Kenji Uryuhara, Yasunari Sakuma, Hirofumi Ichida, Akio Saiura, Koichiro Haruki, Toru Ikegami, Yukihiro Iso, Taku Aoki, Takeshi Takahara, Hideki Yokoo, Yoshifumi Beck, Shigeto Miyagi, Kazuaki Tokodai

**Affiliations:** Department of Gastroenterological and Transplant Surgery, Graduate School of Biomedical and Health Sciences, Hiroshima University, Hiroshima, Japan; Medical Centre for Translational and Clinical Research, Hiroshima University Hospital, Hiroshima, Japan; Department of Biostatistics, Clinical Research Centre in Hiroshima, Hiroshima University Hospital, Hiroshima, Japan; Department of Biostatistics, Clinical Research Centre in Hiroshima, Hiroshima University Hospital, Hiroshima, Japan; Medical Centre for Translational and Clinical Research, Hiroshima University Hospital, Hiroshima, Japan; Department of Surgery and Science, Graduate School of Medical Sciences, Kyushu University, Fukuoka, Japan; Division of Hepato-Biliary-Pancreatic Surgery and Transplantation, Department of Surgery, Graduate School of Medicine, Kyoto University, Kyoto, Japan; Department of Surgery, Graduate School of Biomedical Sciences, Nagasaki University, Nagasaki, Japan; Department of Paediatric Surgery and Transplantation, Graduate School of Medical Sciences, Kumamoto University, Kumamoto, Japan; Division of Artificial Organ and Transplantation, Department of Surgery, Graduate School of Medicine, The University of Tokyo, Tokyo, Japan; Department of Gastroenterological Surgery, Graduate School of Medicine, Dentistry, and Pharmaceutical Sciences, Okayama University, Okayama, Japan; Division of Hepato-Biliary-Pancreatic and Transplantation Surgery, Japanese Red Cross Medical Centre, Tokyo, Japan; Department of Surgery, School of Medicine, Keio University, Tokyo, Japan; Department of Organ Transplantation and General Surgery, Kyoto Prefectural University of Medicine, Kyoto, Japan; Department of Transplantation Surgery, Nagoya University Hospital, Nagoya, Japan; Department of Surgery, School of Medicine, Iwate Medical University, Yahaba, Japan; Department of Hepato-Biliary-Pancreatic Surgery, Institute of Gastroenterology, Tokyo Women's Medical University, Tokyo, Japan; Department of Gastroenterological Surgery, Graduate School of Medicine, Osaka University, Osaka, Japan; Department of Hepato-Biliary-Pancreatic and Breast Surgery, Graduate School of Medicine, Ehime University, Toon, Japan; Division of Gastroenterology, Department of Internal Medicine, Graduate School of Medicine, Kobe University, Kobe, Japan; Organ Transplantation Centre, National Centre for Child Health and Development, Tokyo, Japan; Department of Digestive and Transplantation Surgery, Tokyo Medical University Hachioji Medical Centre, Tokyo, Japan; Division of Gastroenterological, Hepato-Biliary-Pancreatic, Transplantation, and Paediatric Surgery, Department of Surgery, Shinshu University, Matsumoto, Japan; Department of Internal Medicine, Institute of Gastroenterology, Tokyo Women's Medical University, Tokyo, Japan; Department of Gastroenterological, Breast, and Endocrine Surgery, Graduate School of Medicine, Yamaguchi University, Ube, Japan; Department of Diagnostic Pathology, Kyoto University Hospital, Kyoto, Japan; Division of Hepato-Biliary-Pancreatic Surgery, Department of Surgery, Graduate School of Medicine, Kobe University, Kobe, Japan; Department of Gastroenterology and Hepatology, Faculty of Medicine, Saitama Medical University, Moroyama, Japan; Department of Surgery, Osaka International Cancer Institute, Osaka, Japan; Department of Gastroenterological and Transplant Surgery, Graduate School of Biomedical and Health Sciences, Hiroshima University, Hiroshima, Japan

## Abstract

**Background:**

Validating the expanded criteria for living donor liver transplantation for hepatocellular carcinoma using national data is highly significant. The aim of this study was to evaluate the validity of the new Japanese criteria for living donor liver transplantation for hepatocellular carcinoma patients and identify factors associated with a poor prognosis using the Japanese national data set.

**Methods:**

The study population comprised patients who underwent living donor liver transplantation for hepatocellular carcinoma at 37 centres in Japan between 2010 and 2018. In a nationwide survey, the overall survival and recurrence-free survival rates were evaluated based on the new Japanese criteria for applying the 5-5-500 rule when extending the indication beyond the Milan criteria. Prognostic factors within the Japanese criteria were determined using the Cox proportional hazards model.

**Results:**

Patients within (485 patients) and beyond (31 patients) the Japanese criteria exhibited 5-year overall survival rates of 81% and 58% and 5-year recurrence-free survival rates of 77% and 48% respectively. Patients who met the Milan criteria, but not the 5-5-500 rule, had poorer outcomes. Multivariate analysis for 474 patients identified a neutrophil-to-lymphocyte ratio greater than or equal to 5 and a history of hepatectomy as independent risk factors.

**Conclusion:**

This nationwide survey confirms the validity of the Japanese criteria. The poor prognostic factors within the Japanese criteria include a neutrophil-to-lymphocyte ratio greater than or equal to 5 and previous hepatectomy.

## Teaser

This study analysed the most recent nationwide survey (2010–2018) to assess the validity of the new Japanese criteria for living donor liver transplantation for hepatocellular carcinoma patients and identify factors associated with a poor prognosis. The 5-5-500 rule, in conjunction with the Milan criteria, constitutes the Japanese criteria for liver transplantation in patients with hepatocellular carcinoma covered by insurance. However, even within the Japanese criteria, there are specific subgroups of recurrence risks.

## Introduction

With the introduction of the Milan criteria (one nodule less than 5 cm in size or up to three nodules each less than 3 cm in size) in 1996, liver transplantation (LT) emerged as a definitive curative approach for eligible patients with hepatocellular carcinoma (HCC)^1^. However, LT accessibility is limited by the rigidity of the Milan criteria, which has prompted researchers to explore expansion of these criteria, with encouraging results. Nevertheless, the global consensus on LT criteria for HCC remains challenging owing to regional, societal, and national variations in patient enrolment and transplant availability.

Based on a retrospective analysis of a nationwide Japanese cohort survey from 1998 to 2009, Shimamura *et al*.^2^ proposed extended criteria, the 5-5-500 rule (largest nodule size less than or equal to 5 cm in diameter, number of nodules less than or equal to 5, and α-fetoprotein (AFP) value less than or equal to 500 ng/ml), for living donor LT (LDLT) in patients with HCC. Their study demonstrated that patients with HCC meeting the 5-5-500 rule experienced a 7.3% recurrence rate 5 years after LT alongside a 19% increase in LT candidates. The 5-5-500 rule, in conjunction with the Milan criteria, constitutes the Japanese criteria for LT in patients with HCC covered by insurance. However, even within the Japanese criteria, there are specific subgroups of recurrence risks. For instance, patients falling within the Milan criteria, but beyond the 5-5-500 rule, demonstrate lower recurrence-free survival (RFS) than patients falling within the Japanese criteria, with a 5-year RFS rate of approximately 60%.

The aim of this study was to reassess the national data from 2010 to 2018 to validate the new Japanese criteria for LDLT recipients with HCC and elucidate the associated risk factors.

## Methods

### Study design

This multicentre retrospective cohort study enrolled all patients who underwent LDLT for HCC at 37 transplant centres in Japan between January 2010 and December 2018. The inclusion criteria were the presence of at least one HCC nodule on preoperative radiological imaging and histopathological evidence of HCC in the explanted liver. The Japanese criteria met either the Milan criteria^1^ or the 5-5-500 rule (applying the 5-5-500 rule when extending the indication beyond the Milan criteria)^2^. Each institution chose the indication for LT according to the Milan criteria, as well as the Tokyo^3^, Kyoto^4^, and Kyushu^5^ expansion criteria. This retrospective study was conducted in collaboration with the Japanese Liver Transplantation Society, and the Hiroshima University Ethical Committee for Epidemiology approved the study protocol (project number E2021-2778).

### Data collection

Recipient factors included age, sex, BMI, underlying disease, alcohol consumption, smoking history, diabetes history, model for end-stage liver disease (MELD) score, various inflammatory markers (neutrophil-to-lymphocyte ratio (NLR), platelet-to-lymphocyte ratio, lymphocyte-to-monocyte ratio, and Glasgow prognostic score)^6–11^, nutritional scores (prognostic nutritional index and controlling nutritional status score), albumin-bilirubin (ALBI) score^12–15^, and early recurrence after surgery for liver tumour (ERASL) score^16^ at baseline. Donor factors included age, sex, BMI, and blood group compatibility. Surgical factors included operating time, blood loss, and graft-to-recipient weight ratio. Data on preoperative tumour number, maximum tumour diameter, HCC rupture, preoperative treatment, number of preoperative treatments, prior liver resection, AFP levels, des-γ-carboxy prothrombin levels, pathological tumour number, pathological tumour diameter, histological type, vascular invasion, and lymph node metastasis were collected. The Metroticket 2.0 criteria were set as follows: when the AFP level is less than 200 ng/ml, the sum of the size of the largest vital tumour and the number of vital tumour nodules should be less than 7; when the AFP level is 200–400 ng/ml, the sum should be less than 5; and when the AFP level is 400–1000 ng/ml, the sum should be less than 4^17^. Postoperative data included rejection episodes, immunosuppressant regimens, date of last confirmed survival, date of death, date of last confirmed HCC-free status, and date of HCC recurrence.

### Operative procedure and postoperative follow-up

The operative procedure, including graft selection, adhered to standard LDLT protocols and varied among transplant centres. Immunosuppression involves conventional double or triple regimens comprising calcineurin inhibitors (cyclosporine A or tacrolimus) and steroids, with or without adjunctive mycophenolate mofetil or mammalian target of rapamycin inhibitors. HCC recurrence surveillance encompassed regular serum AFP and des-γ-carboxy prothrombin level measurements during follow-up visits (at least once every 3 months) supplemented by contrast-enhanced CT and other relevant imaging modalities for definitive recurrence diagnosis when suspected. Radiological confirmation was primarily performed using CT to confirm HCC recurrence.

### Statistical analysis

The clinical characteristics are summarized using standard descriptive statistics (median (range) or *n* (%)). Overall survival (OS) was defined as the time from surgery to death from any cause or censored at the last follow-up. RFS was defined as the time from surgery to death or recurrence, or censoring at the last follow-up. Survival time was estimated using the Kaplan–Meier method and compared using the log rank test. HRs and confidence intervals were calculated using univariate Cox regression analysis. Multivariate Cox regression models were used to determine the independent prognostic factors after accounting for other factors. Continuous variables were converted into categorical fractions based on conventional or clinical thresholds for Cox regression analyses. All statistical analyses were performed using SAS version 9.4 (SAS Institute, Cary, NC, USA). All *P* values were two-sided and statistical significance was set at *P* < 0.050.

## Results

### Patient characteristics

**Fig. 1:**
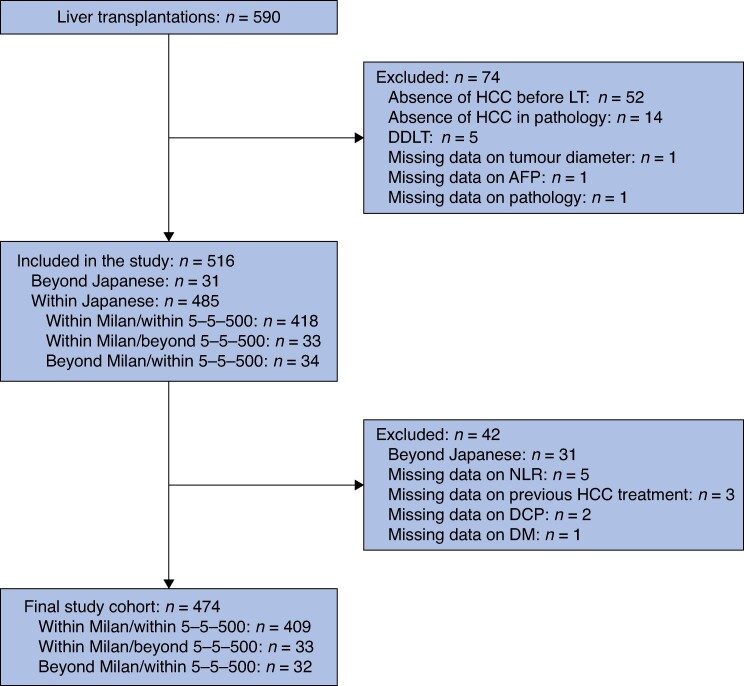
Study flow chart HCC, hepatocellular carcinoma; LT, liver transplantation; DDLT, deceased donor liver transplantation; AFP, α-fetoprotein; NLR, neutrophil-to-lymphocyte ratio; DM, diabetes mellitus.

The clinical characteristics of the patients are summarized in *Table 1*. Among 590 patients undergoing LT at 37 centres across Japan between 2010 and 2018, 516 patients were included in this study. A total of 74 patients were excluded because of the absence of HCC before LT (52 patients), the absence of HCC in pathology (14 patients), receiving a deceased donor LT (5 patients), missing data on tumour diameter (1 patient), missing data on AFP (1 patient), or missing data on pathology (1 patient). Of the 485 patients who met the Japanese criteria, 33 patients satisfied only the Milan criteria, 34 satisfied only the 5–5-500 rule, and 418 satisfied both the Milan criteria and the 5-5-500 rule (*Fig. 1*).

**Table 1:** Patient characteristics (*n* = 516)

Characteristic	Value
Age (years), median (range)	60 (13–75)
Sex	
Male	327 (63)
Female	189 (37)
BMI (kg/m^2^), median (range)	24.53 (15.98–40.35)
Hepatitis B virus	90 (17.44)
Hepatitis C virus	291 (56.4)
Alcoholic	56 (10.85)
NASH	54 (10.47)
Autoimmune hepatitis	22 (4.26)
Donor age (years), median (range)	38 (18–67)
Donor BMI (kg/m^2^), median (range)	22.03 (14.88–31.32)
ABO incompatible	97 (18.8)
MELD score, median (range)	14 (6–37)
NLR, median (range)	2.46 (0.04–84.36)
PLR, median (range)	76.61 (2.31–5000)
LMR, median (range)	2.71 (0–215.29)
GPS (0/1/2), *n*	44/399/73
PNI, median (range)	28.26 (1.2–51.03)
CAR, median (range)	10 (0–2750)
ALBI score, median (range)	−0.89 (−3.16–1.17)
ALBI grade (1 or 2/3), *n*	92/424
ERASL score, median (range)	3.21 (0.29–3221.9)
ERASL grade (1 or 2/3)	285 (55)/231 (45)
AFP (ng/ml), median (range)	14.55 (0–32200)
DCP (mAU/ml), median (range)	72 (2–45153)
Prior HCC treatment	313 (60.66)
Number of HCC pretreatments	1 (0–13)
Prior hepatectomy	50 (9.69)
Tumour number, median (range)	2 (1–99)
Maximum tumour diameter (cm), median (range)	2.0 (0.5–6.8)
Up-to-seven criteria (in/out)	478 (93)/38 (7)
Tokyo criteria (in/out)	492 (95)/24 (5)
Kyoto criteria (in/out)	406 (79)/108 (21)
Kyushu criteria (in/out)	513 (100)/1 (0)
French AFP model (<3/≥3)	462 (90)/54 (10)
Metroticket 2.0 criteria (in/out)	448 (87)/70 (13)
Recipient operating time (min)	777 (150–2440)
Recipient blood loss (ml), median (range)	5120.5 (120–154900)
Pathology tumour number, median (range)	2 (1–278)
Pathology maximum tumour diameter (cm), median (range)	2.0 (0.3–29.0)
Portal vein invasion by pathology	96 (18.6)
Hepatic vein invasion by pathology	25 (4.84)
Hepatic arterial invasion by pathology	2 (0.39)
Bile duct invasion by pathology	11 (2.13)
Pathology vascular invasion	112 (21.71)
Lymph node metastasis	4 (0.78)
RETREAT score, median (range)	2 (1–8)

Values are *n* (%) unless otherwise indicated. NASH, non-alcoholic steatohepatitis; MELD, model for end-stage liver disease; NLR, neutrophil-to-lymphocyte ratio; PLR, platelet-to-lymphocyte ratio; LMR, lymphocyte-to-monocyte ratio; GPS, Glasgow prognostic score; PNI, prognostic nutrition index; CAR, C-reactive protein-to-albumin ratio; ALBI, albumin-bilirubin; ERASL, early recurrence after surgery for liver tumour; AFP, α-fetoprotein; DCP, des-γ-carboxy prothrombin; HCC, hepatocellular carcinoma; RETREAT, risk estimation of tumour recurrence after transplant.

### Clinical outcome

**Fig. 2:**
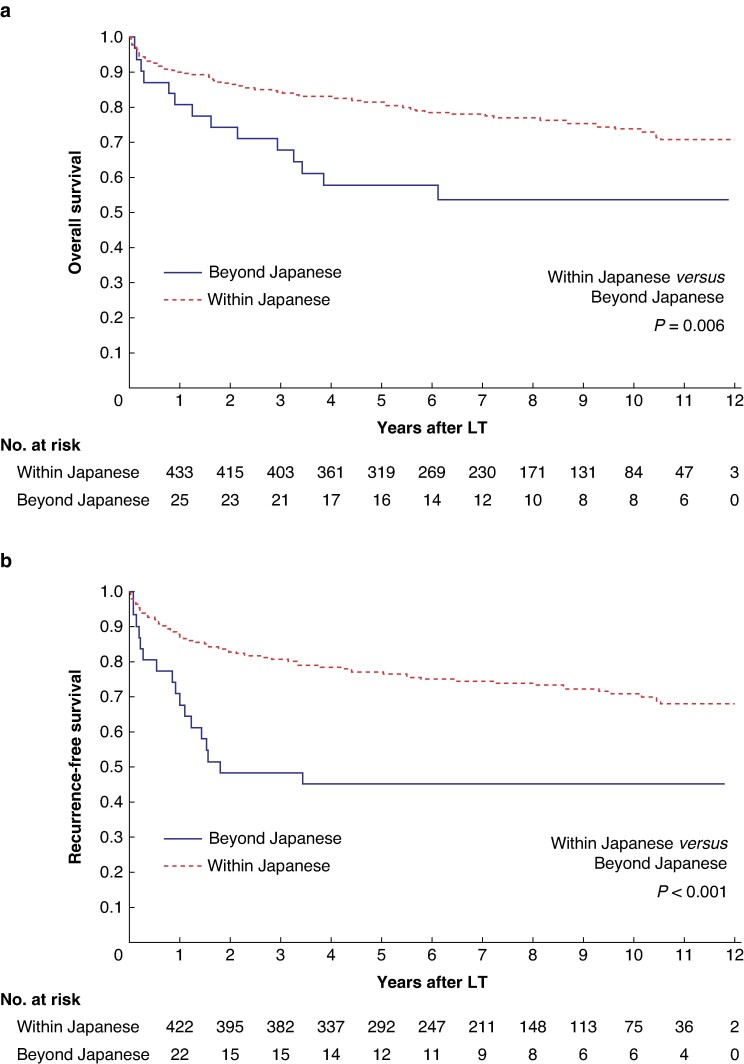
Survival of patients undergoing liver transplantation for hepatocellular carcinoma stratified by the Japanese criteria **a** Overall survival. **b** Recurrence-free survival. LT, liver transplantation.

**Fig. 3:**
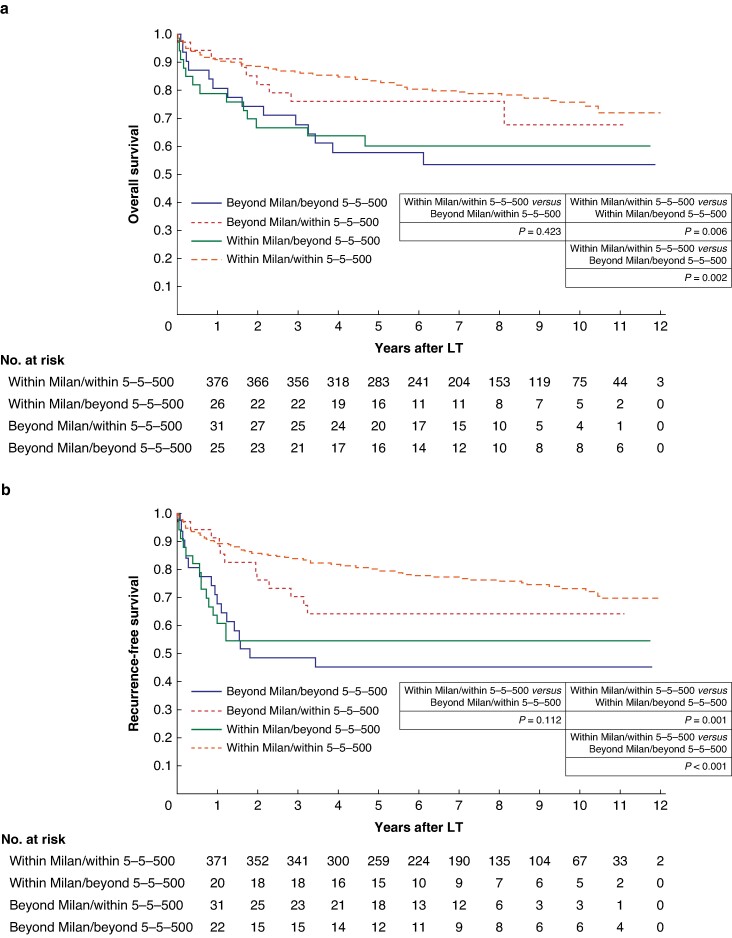
Survival of patients undergoing liver transplantation for hepatocellular carcinoma stratified by the Milan criteria and the 5-5-500 rule **a** Overall survival. **b** Recurrence-free survival. LT, liver transplantation.

The median follow-up duration after the LDLT was 7.78 years (95% c.i. 7.38 to 8.04 years, range 2 days–12.08 years). The 3-year and 5-year OS rates were 87% and 83% respectively. The 3-year and 5-year RFS rates were 84% and 80% respectively. Patients within the Japanese criteria exhibited significantly superior OS compared with patients beyond the Japanese criteria (3-year OS rates of 84% and 68% respectively and 5-year OS rates of 81% and 58% respectively; *P* = 0.006; *Fig. 2a*); similarly, patients within the Japanese criteria exhibited significantly superior RFS compared with patients beyond the Japanese criteria (3-year RFS rates of 81% and 48% respectively and 5-year RFS rates of 77% and 45% respectively; *P* < 0.001; *Fig. 2b*). Subsequently, the patients within the Japanese criteria were categorized into three groups for analysis: within the Milan criteria and the 5-5-500 rule; beyond the Milan criteria and within the 5-5-500 rule; and within the Milan criteria and beyond the 5-5-500 rule. The 3-year and 5-year OS rates were significantly lower in the group within the Milan criteria and beyond the 5-5-500 rule than in the group within the Milan criteria and the 5-5-500 rule (*Fig. 3a*); similarly, the 3-year and 5-year RFS rates were significantly lower in the group within the Milan criteria and beyond the 5-5-500 rule than in the group within the Milan criteria and the 5-5-500 rule (*Fig. 3b*). Furthermore, an analysis was performed comparing the pathological outcomes of the Milan criteria and the 5-5-500 rule (*Fig. S1*). The outcomes were closely aligned based on the preoperative imaging and pathological evaluations. Moreover, the analytical approach described in the Metroticket 2.0 model^17^ (which is widely utilized in Europe and the USA) was implemented in the current cohort. The results are shown in *Fig. S2*. The survival curves strongly corresponded to the Japanese criteria.

### Prognostic factors

Of the 516 patients, an independent analysis was conducted to identify risk factors associated with RFS in 474 patients who met the Japanese criteria, excluding 31 patients beyond the Japanese criteria and 11 with insufficient data (*Fig. 1*). A detailed description of the number of tumours and the maximum tumour diameter according to the Japanese criteria is presented in *Table S1*. Univariate analysis revealed that a high NLR, elevated AFP levels, ALBI grade, ERASL grade, prior hepatectomy, French AFP model, and Kyoto criteria were significant risk factors for poor RFS (*Table 2*). In the multivariate analysis, independent prognostic factors for RFS included an NLR greater than or equal to 5 (HR 1.832 (95% c.i. 1.215 to 2.764); *P* = 0.004) and previous hepatectomy (HR 1.952 (95% c.i. 1.127 to 3.279); *P* = 0.017; *Table 2*). To elucidate why hepatectomy emerged as an adverse prognostic factor, while preoperative treatment itself did not, a comparison was conducted between the hepatectomy group and the non-hepatectomy group within the preoperative treatment subgroup (*Table S2*). In the hepatectomy group, markers such as the lymphocyte-to-monocyte ratio, the Glasgow prognostic score, and the prognostic nutritional index were higher and the ALBI score was lower. The hepatectomy group had significantly lower MELD scores, had a greater proportion of preoperative treatment, and exhibited lesser pathological vascular invasion. This suggests that the hepatectomy group did not necessarily have more advanced cirrhosis and does not indicate a higher degree of tumour malignancy. The lack of difference in the surgical time and blood loss suggests that post-hepatectomy adhesions did not complicate the surgical procedure or adversely affect the postoperative recovery.

**Table 2:** Univariate and multivariate analysis of prognostic factors within the Japan criteria

Factors	Univariate			Multivariate		
	HR	95% CI	*P*	HR	95% CI	*P*
Recipient age <60 years	1.38	0.97–1.97	0.074	–	–	–
Donor age ≥50 years	1.26	0.86–1.83	0.234	–	–	–
Sex (male/female)	1.31	0.90–1.91	0.165	–	–	–
Recipient BMI <25	0.96	0.67–1.27	0.820	–	–	–
HBV	0.74	0.45–1.22	0.235	–	–	–
HCV	1.36	0.94–1.95	0.102	–	–	–
Diabetes Mellites	1.08	0.72–1.62	0.709	–	–	–
NLR ≥ 5	1.63	1.09–2.43	0.018	1.83	1.22–2.76	0.004
PLR > 150	1.38	0.87–2.21	0.175	–	–	–
GPS 2/0 or 1	1.42	0.88–2.30	0.149	–	–	–
PNI ≥ 40	1.70	0.79–3.64	0.176	–	–	–
CAR > 26	1.12	0.74–1.69	0.596	–	–	–
MELD ≥ 20	0.95	0.60–1.60	0.832	–	–	–
AFP > 500 (ng/mL)	2.37	1.38–4.07	0.002	1.81	0.81–4.07	0.149
DCP ≥ 1000 (mAU/mL)	1.63	0.98–2.73	0.060	–	–	–
CRP ≥ 1 (mg/dL)	1.53	0.98–2.38	0.064	–	–	–
ALBI Grade 1 or 2/3	1.53	1.01–2.30	0.044	1.46	0.94–1.96	0.090
ERASL Grade 3/1 or 2	1.44	1.01–2.05	0.041	1.33	0.91–1.96	0.145
ABO incompatibility	0.97	0.60–1.55	0.885	–	–	–
Prior treatment of HCC	1.34	0.93–1.94	0.118	–	–	–
Prior hepatectomy for HCC	1.88	1.12–3.13	0.016	1.95	1.13–3.38	0.017
Up-to-seven criteria out	1.95	0.72–5.28	0.190	–	–	–
AFP-model ≥3	2.12	1.20–3.77	0.010	1.26	0.54–2.96	0.596
Kyoto criteria	1.61	1.07–2.40	0.021	1.52	1.00–2.31	0.050

AFP, alpha-fetoprotein; ALBI grade, albumin-bilirubin grade; BMI, body mass index; CAR, C-reactive protein-to-albumin ratio; CRP, C-reactive protein; DCP, des-gamma-carboxyprothrombin; ERASL, early recurrence after surgery for liver tumor; GPS, Glasgow Prognostic Score; HBV, hepatitis B; HCV, hepatitis C; MELD, The Model of End-stage Liver Disease; NLR, neutrophil-to-lymphocyte ratio; PLR, platelet-to-lymphocyte ratio.

## Discussion

This study used nationwide data from Japan to validate the efficacy of the Japanese criteria for LDLT in patients with HCC. The study population comprised patients undergoing LT performed in Japan, while the pre-revision insurance coverage criteria (Milan criteria) were effective. The fact that LT had to be performed as a self-funded treatment in patients who did not meet the Milan criteria likely affected the distribution of patients outside these criteria. Therefore, assuming that insurance coverage during this interval had expanded to the Japanese criteria, 7.54% of patients (34 patients who did not meet the Milan criteria, but met the Japanese criteria) would have benefited from insurance coverage. The outcomes of patients who met the Japanese criteria alone (falling outside the Milan criteria, but within the 5-5-500 rule) were comparable to those of patients who met the established Milan criteria (*Fig. S3*). A previous study that included 31.2% (301/964) of patients who did not meet the Milan criteria estimated that 19% of patients would benefit from insurance coverage due to the new Japanese criteria^2^. In this study of patients, with LT conducted under stricter insurance coverage, only 12.6% (65/516) of the patients did not meet the Milan criteria.

Hence, the percentage of patients benefiting from insurance coverage decreased with the application of the Japanese criteria. However, the percentages of patients who met the 5-5-500 rule among those who did not meet the Milan criteria were 42.5% (128/301) in a previous study and 52.3% (34/65) in the present study, which are comparable. These findings suggest that the practical application of the Japanese criteria will allow a certain percentage of patients outside the Milan criteria to undergo LT with the expectation of an acceptable prognosis under insurance coverage; however, the number of such patients cannot be predicted by retrospective studies, such as previous studies and the present study. Despite these limitations, this study demonstrated that the 5-5-500 rule identifies patients outside the Milan criteria who are at low risk of recurrence and the validity of the Japanese criteria that combine the Milan criteria with the 5-5-500 rule. The clinical efficacy of the Japanese criteria in predicting the post-LT prognosis for HCC was found to be nearly equivalent to that of the internationally recognized Metroticket 2.0 criteria^17^ (*Fig. S2*). Nonetheless, considering that the subset of patients who did not fulfill the Japanese criteria was smaller than that of those who did not meet the Metroticket 2.0 criteria, one can infer that adopting the Japanese criteria as an expanded benchmark could potentially lead to the inclusion of more extensive patient demographics.

Another noteworthy result of this study was the identification of a subgroup of patients with LT with risk factors for recurrence, even among those who met the Japanese criteria. It was found that patients who exceeded the 5-5-500 criteria, even within the Milan criteria (equivalent to AFP greater than 500 ng/ml, even within the Milan criteria), had a low RFS rate. In addition, the presence of an NLR greater than or equal to 5 and a history of hepatectomy before LDLT were also identified as unfavourable prognostic factors for patients within the Japanese criteria. Thus, in addition to the physical burden of tumours, that is the number and size of tumours, the risk group for recurrence could be predicted more efficiently by employing biological markers that may reflect the malignant potential or inflammatory status of the host. In this study, the AFP value before LT showed a clear relationship with the recurrence rate; however, the absolute AFP value and the dynamic changes during the waiting interval for LT have also been reported as prognostically relevant factors^18–20^.

NLR is a recognized biomarker of systemic inflammation and has been implicated as an indicator of graft failure and rejection in organ transplantation^21,22^. Moreover, it is widely used as a prognostic marker for solid tumours^23^. Neutrophils contribute to the release of vascular endothelial growth factor (VEGF) and elevated VEGF levels are associated with cancer invasion and metastasis^24^. Decreased lymphocyte levels are associated with impaired tumour immunity^25^. Several studies have explored the role of NLR in LT in patients with HCC. For instance, a study involving 189 patients who underwent LT for hepatitis B virus-related liver cancer identified a high NLR as a predictor of survival and RFS^8^. Similarly, in a cohort of 190 LDLT patients, a high NLR emerged as an independent prognostic factor for RFS^12^. Conversely, other reports have indicated that a high NLR is associated with HCC vascular invasion and poorly differentiated explanted livers, but not with the post-transplant prognosis^26^. Although it has been pointed out that the cut-off value of NLR and the timing of blood collection may affect the results, the reliability of this study is considered to be high because data from the most recent LT were collected from multiple centres. Consequently, the findings, which highlight the use of elevated NLR values in national data, are of substantial significance.

In this study, the effect of preoperative HCC treatment on the prognosis was insignificant. In contrast, prior hepatectomy has emerged as an independent unfavourable prognostic factor. Pre-transplant therapy for HCC can be categorized into three groups: tumour down-staging to reduce tumour size; maintenance therapy to stabilize tumour size; and salvage LT for tumour recurrence. Although the overall outcome of LT did not differ significantly with or without preoperative HCC treatment, an increased number of treatments before transplantation may negatively impact the outcome^27–29^. However, the findings of the present study revealed no significant differences in the number of preoperative treatments. Prior hepatectomy is primarily associated with salvage LT. A recent meta-analysis demonstrated that salvage LT was associated with lower 5-year OS and RFS rates than primary LT; however, complication rates did not significantly differ^30^. Nonetheless, favourable outcomes of salvage LT have been reported by defining indications based on tumour markers and size^31,32^. Moreover, previous upper abdominal surgery has been linked to an adverse prognosis after LT, potentially due to surgical challenges and postoperative complications associated with adhesions^33^. It is peculiar that hepatectomy alone emerged as an adverse prognostic factor, despite preoperative treatment itself not affecting the prognosis. Subgroup analysis revealed that the hepatectomy group did not necessarily have higher MELD scores or greater tumour malignancy. However, differences in inflammatory and nutritional status were observed, suggesting that patient conditions not reflected by numerical values may have adversely impacted the host’s antitumour capabilities. Given the retrospective nature of this study, detailed data for each patient were not available and the present analysis is likely to be limited.

This study is limited by its retrospective design. Owing to the retrospective and multicentre nature of this study, data collection was limited, leading to numerous omissions regarding preoperative treatment and its outcomes. Nonetheless, the importance of this study lies in its validation of previously established national data and confirmation of the validity of the Japanese criteria and risk stratification. Another limitation was the lack of data on postoperative immunosuppressive drug use, hepatitis C virus treatment status, HCC recurrence site, and duration of HCC disease before transplantation. These factors could potentially influence HCC recurrence and should be considered in future studies, particularly considering the multi-institutional nature of this study.

The findings of this validation study provide further evidence supporting the appropriateness of the Japanese criteria. Consistent with previous cohorts, patients who met the Milan criteria, but exceeded the 5-5-500 rule, exhibited an unfavourable prognosis. In contrast, prior hepatectomy and an elevated NLR were independent poor prognostic factors. These observations underscore the importance of considering these factors when evaluating and managing patients undergoing LT for HCC.

## Collaborators

**Japanese Liver Transplantation Society**

Shugo Mizuno (Hepatobiliary Pancreatic and Transplant Surgery, Mie University Graduate School of Medicine, Tsu, Japan); Satoshi Kuboki, Masayuki Otsuka (Department of General Surgery, Chiba University, Chiba, Japan); Takashi Kobayashi, Toshifumi Wakai (Digestive and General Surgery Niigata University, Niigata, Japan); Teruhide Ishigame, Shigeru Marubashi (Department of Hepato-Biliary-Pancreatic and Transplant Surgery, Fukushima Medical University, Fukushima, Japan); Masaaki Watanabe, Akinobu Taketomi (Department of Gastroenterological Surgery I, Hokkaido University, Sapporo, Japan); Shinichi Nakamuma, Shintaro Yagi (Department of Hepato-Biliary-Pancreatic Surgery and Transplantation, Kanazawa University, Kanazawa, Japan); Taichi Wakiya, Keinosuke Ishido (Department of Gastroenterological Surgery and Paediatric Surgery, Hirosaki University Graduate School of Medicine, Hirosaki, Japan); Yu Sawada, Itaru Endo (Department of Gastroenterological Surgery, Yokohama City University Graduate School of Medicine, Yokohama, Japan); Yuichi Goto, Toru Hisaka (Department of Surgery, Kurume University, Kurume, Japan); Kenji Uryuhara (Department of Surgery, Kobe City Medical Centre General Hospital, Kobe, Japan); Yasunari Sakuma (Department of Transplant Surgery, Jichi Medical University, Shimotsuke, Japan); Hirofumi Ichida, Akio Saiura (Department of Hepatobiliary-Pancreatic Surgery, Juntendo University School of Medicine, Tokyo, Japan); Koichiro Haruki, Toru Ikegami (Division of Hepatobiliary and Pancreas Surgery, Department of Surgery, The Jikei University School of Medicine, Tokyo, Japan); Yukihiro Iso, Taku Aoki (Department of Hepato-Biliary-Pancreatic Surgery, Dokkyo Medical University, Mibu, Japan); Takeshi Takahara (Department of Surgery, Fujita Health University, Toyoake, Japan); Hideki Yokoo (Department of Hepatobiliary, Pancreatic and Transplant Surgery, Asahikawa Medical University, Asahikawa, Japan); Yoshifumi Beck (Department of Hepato-Biliary-Pancreatic Surgery and Paediatric Surgery, Saitama Medical Centre, Saitama Medical University, Moroyama, Japan); Shigeto Miyagi, Kazuaki Tokodai (Department of Surgery, Tohoku University Graduate School of Medicine, Sendai, Japan).

## Supplementary Material

zrae079_Supplementary_Data

## Data Availability

The data supporting the findings of this study are available from the corresponding author upon reasonable request.
