# Supplementary material for: Japanese living donor liver transplantation criteria for hepatocellular carcinoma: nationwide cohort study
Source: BJS Open. 2024 Aug 2;8(4):zrae079. doi: 10.1093/bjsopen/zrae079 (PMC11295212; doi:10.1093/bjsopen/zrae079)
Supplement: zrae079_Supplementary_Data [file zrae079_supplementary_data.docx]

**Title**

**Japan Living Donor Liver Transplant Criteria for Hepatocellular Carcinoma: A Nationwide Cohort Study**

Authors:

Masahiro Ohira, MD, PhD^1,2^, Gaku Aoki, PhD^3^, Yasushi Orihashi, PhD^3^, Kenichi Yoshimura PhD^2^, Takeo Toshima MD, PhD^4^, Etsuro Hatano MD, PhD^5^, Susumu Eguchi MD, PhD^6^, Taizo Hibi MD, PhD^7^, Kiyoshi Hasegawa MD, PhD^8^, Yuzo Umeda MD, PhD^9^, Takuya Hashimoto MD, PhD^10^, Yasushi Hasegawa MD, PhD^11^, Shuji Nobori MD, PhD^12^, Yasuhiro Ogura MD, PhD^13^, Hiroyuki Nitta MD,PhD^14^, Hiroto Egawa MD, PhD^15^, Hidetoshi Eguchi MD, PhD^16^, Yasutsugu Takada MD, PhD^17^, Yoshihide Ueda MD, PhD^18^, Mureo Kasahara MD, PhD^19^, Shigeyuki Kawachi MD, PhD^20^, Yuji Soejima MD, PhD^21^, Katsutoshi Tokushige MD, PhD^22^, Hiroaki Nagano MD, PhD^23^, Hironori Haga MD, PhD^24^, Takumi Fukumoto MD, PhD^25^, Satoshi Mochida MD, PhD^26^, Koji Umeshita MD, PhD^27^, Hideki Ohdan MD, PhD^1^ & on behalf of the Japanese Liver Transplantation Society

Affiliations:

^1^ Department of Gastroenterological and Transplant Surgery, Graduate School of Biomedical and Health Sciences, Hiroshima University, Hiroshima, Japan

^2^ Medical Center for Translational and Clinical Research, Hiroshima University Hospital, Hiroshima, Japan

^3^ Department of Biostatistics, Clinical Research Center in Hiroshima, Hiroshima University Hospital, Hiroshima, Japan

^4^ Department of Surgery and Science, Graduate School of Medical Sciences, Kyushu University, Fukuoka, Japan

^5^ Division of Hepato-Biliary-Pancreatic Surgery and Transplantation, Department of Surgery, Graduate School of Medicine, Kyoto University, Kyoto, Japan

^6^ Department of Surgery, Nagasaki University Graduate School of Biomedical Sciences, Nagasaki, Japan

^7^ Department of Pediatric Surgery and Transplantation, Kumamoto University Graduate School of Medical Sciences, Kumamoto, Japan

^8^ Artificial Organ and Transplantation Division, Department of Surgery, Graduate School of Medicine, The University of Tokyo, Tokyo, Japan

^9^ Department of Gastroenterological Surgery, Okayama University Graduate School of Medicine, Dentistry, and Pharmaceutical Sciences, Okayama, Japan

^10^ Division of Hepato-Biliary-Pancreatic and Transplantation Surgery, Japanese Red Cross Medical Center, Tokyo, Japan

^11^ Department of Surgery, Keio University School of Medicine, Tokyo, Japan

^12^ Department of Organ Transplantation and General Surgery, Kyoto Prefectural University of Medicine, Kyoto, Japan

^13^ Department of Transplantation Surgery, Nagoya University Hospital, Nagoya, Japan

^14^ Department of Surgery, Iwate Medical University School of Medicine, Yahaba, Japan

^15^ Department of Hepatobiliary-pancreatic Surgery, Institute of Gastroenterology, Tokyo Women's Medical University, Tokyo, Japan

^16^ Department of Gastroenterological Surgery, Osaka University Graduate School of Medicine, Osaka, Japan

^17^ Department of Hepato-Biliary-Pancreatic and Breast Surgery, Ehime University Graduate School of Medicine, Toon, Japan

^18^ Department of Internal Medicine, Division of Gastroenterology, Kobe University Graduate School of Medicine, Kobe, Japan

^19^ Organ Transplantation Center, National Center for Child Health and Development, Tokyo, Japan

^20^ Department of Digestive and Transplantation Surgery, Tokyo Medical University Hachioji Medical Center, Tokyo, Japan

^21^ Department of Surgery, Division of Gastroenterological, Hepato-Biliary-Pancreatic, Transplantation and Pediatric Surgery, Shinshu University, Matsumoto, Japan

^22^ Institute of Gastroenterology, Department of Internal Medicine, Tokyo Women's Medical University, Tokyo, Japan

^23^ Department of Gastroenterological, Breast and Endocrine Surgery, Yamaguchi University Graduate School of Medicine, Ube, Japan

^24^ Department of Diagnostic Pathology, Kyoto University Hospital, Kyoto, Japan

^25^ Department of Surgery, Division of Hepato-Biliary-Pancreatic Surgery, Kobe University Graduate School of Medicine, Hyogo, Japan

^26^ Department of Gastroenterology & Hepatology, Faculty of Medicine, Saitama Medical University, Moroyama, Japan

^27^ Department of Surgery, Osaka International Cancer Institute, Osaka, Japan

**Corresponding author.** Hideki Ohdan M.D., Ph.D.

Department of Gastroenterological and Transplant Surgery, Graduate School of Biomedical and Health Sciences, Hiroshima University, 1-2-3 Kasumi, Minami-ku, Hiroshima city, Hiroshima, Japan 734-8551 E-mail: hohdan@hiroshima-u.ac.jp Tel: 81-82-257-5220 Fax: 81-82-257-5224

**Supplementary Materials - Index**

| **Supplementary Figures and Tables** |  |
| --- | --- |
| Supplementary Figure 1 | *page 3* |
| Supplementary Figure 2 | *page 4* |
| Supplementary Figure 3 | *page 5* |
| Supplementary Table 1 | *page 6* |
| Supplementary Table 2 | *page 7* |

**Supplementary Figures and Tables**

**Supplementary Figure 1**

**
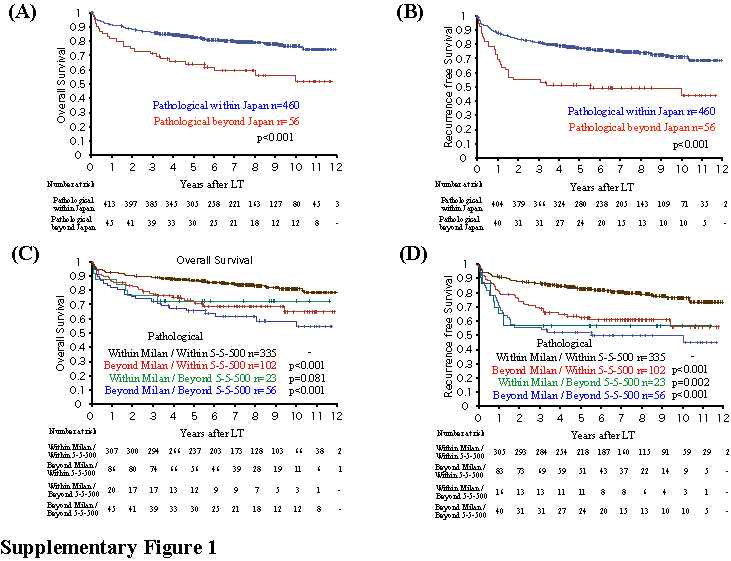
**

Overall survival (A) and recurrence-free survival (B) of patients undergoing liver transplantation for hepatocellular carcinoma stratified by the Japan criteria according the pathological findings. Overall survival (C) and recurrence-free survival (D) of patients undergoing liver transplantation for hepatocellular carcinoma stratified by the Milan criteria and the 5-5-500 rule according to the pathological findings.

**Supplementary Figure 2**

**
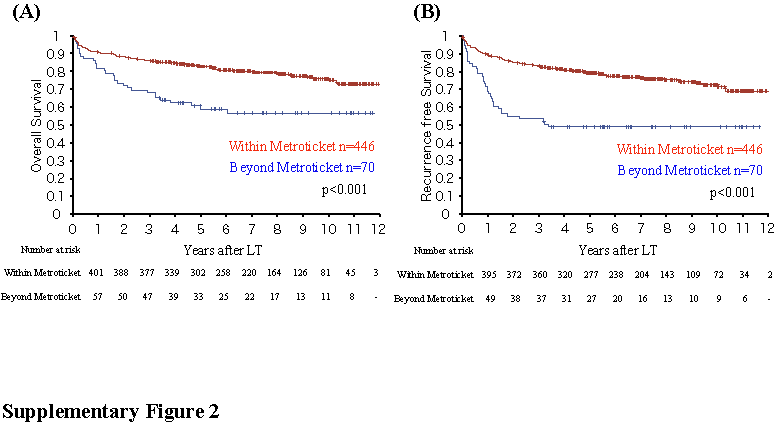
**

Overall survival (A) and recurrence-free survival (B) of patients undergoing liver transplantation for hepatocellular carcinoma stratified by the MetroTicket 2.0.

**Supplementary Figure 3**

**
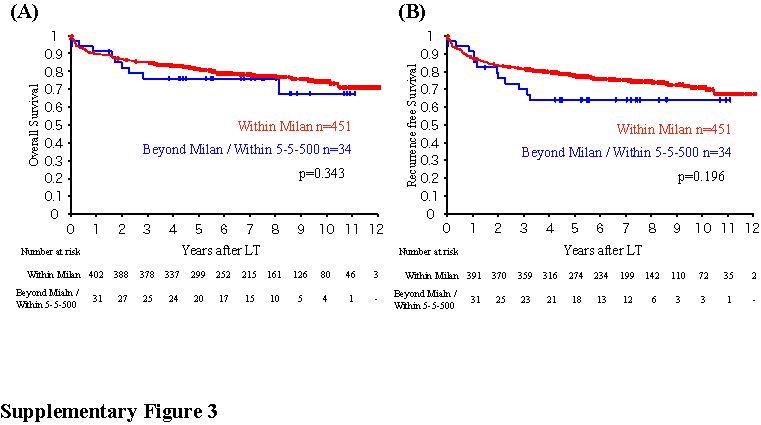
**

Overall survival (A) and recurrence-free survival (B) of patients undergoing liver transplantation for hepatocellular carcinoma between within Milan criteria and beyond Milan/within 5-5-500.

**Supplementary TABLE 1. Number of Tumors and Maximum Tumor Diameter of within the Japan criteria**

| Factors | N=474 |
| --- | --- |
| Tumor number: |  |
| 1 | 247 (52.1%) |
| 2 | 119 (25.1%) |
| 3 | 87 (18.3%) |
| 4 | 15 (3.2%) |
| 5 | 6 (1.3%) |
|  |  |
| Maximum tumor diameter: |  |
| 3cm or more | 81 (17.1%) |
| Less than 3cm | 393 (82.9%) |
|  |  |
| Tumor number and maximum tumor diameter: |  |
| 3 tumors or more and 3cm or more | 16 (3.4%) |
| Others | 458 (96.6%) |
|  |  |

**Supplementary TABLE 2. Comparison of prior hepatectomy and pre-treatment without hepatectomy groups**

| Factors | Prior hepatectomy n=40 | Pre-treatment without hepatectomy n=241 | p value |
| --- | --- | --- | --- |
| Age (year) | 59 (47-71) | 60 (34-72) | 0.1882 |
| Gender (M/F) | 28/12 | 164/77 | 0.8060 |
| BMI (kg/m^2^) | 24.4 (19.1-28.5) | 24.6 (16.0-36.4) | 0.2774 |
|  |  |  |  |
| Donor Age (year) | 47.5 (20-64) | 37 (19-67) | 0.2464 |
| Donor BMI (kg/m^2^) | 22.1 (16.8-28.2) | 22.4 (14.9-31.3) | 0.4770 |
| ABO incompatible | 6 (15.0%) | 40 (16.6%) | 0.8004 |
|  |  |  |  |
| MELD | 12 (6-23) | 13 (6-31) | 0.0228 |
| NLR | 2.1 (0.6-9.9) | 2.4 (0.0-41.5) | 0.1684 |
| PLR | 75.2 (8.9-365.5) | 75.4 (2.3-5000) | 0.2101 |
| LMR | 3.4 (1.1-71.0) | 2.6 (0.1-215.4) | 0.0123 |
| GPS (0/1/2) | 3/27/10 | 28/194/19 | 0.0041 |
| PNI | 31.6 (4.7-46.8) | 28.4 (1.2-51.0) | 0.0120 |
| ALBI score | -1.3 (-2.8-1.2) | -0.9 (-3.2-1.0) | 0.0070 |
| ERASL score | 2.8 (1.2-540.5) | 3.1 (0.3-1286.4) | 0.2406 |
|  |  |  |  |
| AFP (ng/ml) | 9.8 (2.0-5384) | 12.9 (0-12852) | 0.7670 |
| DCP (mAU/mL) | 100.5 (10-18420) | 57.0 (2.0-19354) | 0.2691 |
|  |  |  |  |
| Number of HCC pretreatments | 3 (1-7) | 2 (1-13) | 0.0157 |
| Tumor number | 1 (1-3) | 2 (1-5) | 0.2222 |
| Max tumor diameter (cm) | 1.7 (0.5-4.0) | 2.0 (0.5-4.9) | 0.1206 |
|  |  |  |  |
| Operation time (minutes) | 794 (150-1270) | 792 (342-1912) | 0.9454 |
| Blood loss (ml) | 5911 (920-63680) | 5200 (150-154900) | 0.4246 |
|  |  |  |  |
| Pathology tumor number | 2 (1-22) | 2 (1-36) | 0.8303 |
| Pathology max tumor diameter (cm) | 2.0 (0.5-20.0) | 2.1 (0.3-29.0) | 0.3401 |
| vp | 3 (7.5%) | 49 (20.3%) | 0.0529 |
| vv | 0 (0%) | 13 (5.4%) | 0.1326 |
| va | 0 (0%) | 0 (0%) | 1.0000 |
| b | 0 (0%) | 5 (2.1%) | 0.3580 |
| Pathology vascular invasion | 3 (7.5%) | 59 (24.5%) | 0.0165 |

Continuous variables are expressed as medians (ranges). Qualitative variables are expressed as numbers (%). Abbreviations: AFP, alpha-fetoprotein; ALB, albumin; ALBI score, albumin-bilirubin score; b, bile duct invasion by pathology; BMI, body mass index; DCP, des-gamma-carboxyprothrombin; ERASL, Early Recurrence After Surgery for Liver tumor; GPS, Glasgow Prognostic Score; HCC, hepatocellular carcinoma; LMR, Lymphocyte/Monocyte Ratio; MELD, Model For End-Stage Liver Disease; NLR, Neutrophil/Lymphocyte Ratio; PLR, Platelet/Lymphocyte Ratio; PNI, Prognostic Nutrition Index; va, hepatic arterial invasion by pathology; vp, portal vein invasion by pathology; vv, hepatic vein invasion by pathology
